# Efficacy and Safety of Dry Powder Antibiotics: A Narrative Review

**DOI:** 10.3390/jcm12103577

**Published:** 2023-05-20

**Authors:** David de la Rosa-Carrillo, Guillermo Suárez-Cuartín, Oriol Sibila, Rafael Golpe, Rosa-María Girón, Miguel-Ángel Martínez-García

**Affiliations:** 1 Respiratory Department, Hospital de la Santa Creu I Sant Pau, 08041 Barcelona, Spain; 2 Respiratory Department, Hospital de Bellvitge, 08907 L’Hospitalet de Llobregat, Spain; gsuarezcuartin@gmail.com; 3 Respiratory Department, Hospital Clínic i Provincial, 08036 Barcelona, Spain; osbila@clinic.cat; 4 Respiratory Department, Hospital Lucus Augusti, 27003 Lugo, Spain; rafagolpe@gmail.com; 5 Respiratory Department, Hospital de la Princesa, 28006 Madrid, Spain; rmgiron@gmail.com; 6 Respiratory Department, Hospital La Fe, 46026 Valencia, Spain

**Keywords:** antibiotics, inhalation drug, *Pseudomonas aeruginosa*, chronic bronchial infection, bronchiectasis, cystic fibrosis, dry powder antibiotics

## Abstract

The use of inhaled antibiotics was initially almost exclusively confined to patients with cystic fibrosis (CF). However, it has been extended in recent decades to patients with non-CF bronchiectasis or chronic obstructive pulmonary disease who present with chronic bronchial infection by potentially pathogenic microorganisms. Inhaled antibiotics reach high concentrations in the area of infection, which enhances their effect and enables their long-term administration to defeat the most resistant infections, while minimizing possible adverse effects. New formulations of inhaled dry powder antibiotics have been developed, providing, among other advantages, faster preparation and administration of the drug, as well as avoiding the requirement to clean nebulization equipment. In this review, we analyze the advantages and disadvantages of the different types of devices that allow the inhalation of antibiotics, especially dry powder inhalers. We describe their general characteristics, the different inhalers on the market and the proper way to use them. We analyze the factors that influence the way in which the dry powder drug reaches the lower airways, as well as aspects of microbiological effectiveness and risks of resistance development. We review the scientific evidence on the use of colistin and tobramycin with this type of device, both in patients with CF and with non-CF bronchiectasis. Finally, we discuss the literature on the development of new dry powder antibiotics.

## 1. Introduction

Although the use of aerosol therapy as a treatment for respiratory diseases dates back to ancient civilizations, it has now become the modality of choice for the administration of drugs that require direct action on the lower airways, as this type of therapy produces greater local deposition and is associated with fewer secondary effects than systemic administration. The presence of chronic bronchial inflammation [1,2,3] and infection [4,5] in both steady-state and exacerbation periods [6,7,8] of some airway diseases such as bronchiectasis, cystic fibrosis or COPD were determined as hard to treat and have been associated with a poor prognosis. 

The use of antibiotics by direct instillation in the tracheobronchial tree or by nebulization began in the 1940s in patients with cystic fibrosis (CF) and other pathologies accompanied by chronic and/or repeated bronchial infections [9,10]. The nebulized administration of antibiotics became widespread in the early 1980s in patients with CF, because the published results of the first trials showed high concentrations in the area of infection, enhancing their effect and enabling their long-term administration to defeat the most resistant infections, while minimizing possible adverse effects.

The use of inhaled antibiotics has been extended to patients with non-CF bronchiectasis (hereafter bronchiectasis) or chronic obstructive pulmonary disease (COPD) who present with chronic bronchial infection (CBI) by potentially pathogenic microorganisms (PPM) [11]. However, there is still a lack of scientific evidence to demonstrate their effectiveness and safety in this population, so their formal indication is restricted to CBI due to *Pseudomonas aeruginosa* in CF patients. Nevertheless, several bronchiectasis guidelines recommend the use of inhaled antibiotics in patients suffering from IBC and frequent infectious exacerbations [12,13,14]. There are no recommendations for their use in the current guidelines for COPD, however, owing to the limited data on their effectiveness in this disease. Nevertheless, there is a consensus document that proposes the administration of inhaled antibiotics in patients with COPD and CBI due to *P. aeruginosa* or other particularly virulent PPM [15].

In the last few decades, new formulations of inhaled antibiotics have been developed in patients with CF and bronchiectasis. Until recently, the only way to administer inhaled antibiotic treatment was through nebulizers, which is time-consuming and requires rigorous maintenance of the device after each use. Moreover, nebulizers are difficult to transport and require a power supply for operation. In recent years, other antibiotic devices and formulations have been designed to allow administration by inhalation in the form of a dry powder [16,17]. These provide multiple advantages over nebulizers, such as their small size, speed of administration, few cleaning requirements and disposability, thereby minimizing the risk of microbial contamination.

This article aims to review the different types of inhalation devices, with special emphasis on dry powder inhalers (DPIs). We will describe their general characteristics, the way in which they facilitate the arrival of the drug to the lower airway and microbiological aspects of interest. We will also analyze the scientific evidence regarding the use of colistin and tobramycin in this type of devices in patients with CF and bronchiectasis. Finally, we will discuss the literature on the development of new dry powder antibiotics.

## 2. Nebulized Antibiotics: General Characteristics and Modalities

Aerosol therapy is based on the administration of substances in the form of stable particles, either solid or liquid, suspended in a gas (aerosol), by inhalation. There are three types of inhalation devices: the pressurized metered dose inhaler (pMDI), DPIs and nebulizers. In nebulizers, the drug is in suspension or in an aqueous solution and is sprayed as small droplets that can be inhaled by different methods [18]. Nebulizers consist of a misting chamber and an energy source. They come in three types: ultrasonic, jet (also called pneumatic or air jet) and mesh (also called electronic) (Table 1). Inhaling the spray generated from the chamber requires an interface, which can be a mouthpiece, a nasobuccal mask, a T-connection to the trachea, a tracheal mask or a nasal hairpin.

### 2.1. Ultrasonic Nebulizers

These work by means of the high-frequency vibration of a piezoelectric crystal, which produces oscillations in the liquid, leading to nebulization. The greater the vibration, the smaller the generated particles will be. These devices are not appropriate for the nebulization of antibiotics or other drugs in suspension, such as corticosteroids and DNase, since part of the waves generated by the vibration dissipate as heat, which can affect the drug’s stability [20,21].

### 2.2. Jet Nebulizers

In this case, the aerosol is generated by colliding with a jet of gas in the nebulizer chamber: as the speed of the fluid increases, its pressure decreases (the Venturi Effect) and the resulting negative pressure causes the liquid to rise (the Bernoulli Effect) and hit a percussion system, which fragments it into numerous small droplets. The larger droplets return to the reservoir, while the smaller ones can be inhaled. Jet nebulizers can use a mechanical air or oxygen compressor as a source of energy. A compressor is usually most suited to use in a patient’s home, due to its easy maintenance. The greater the flow, the smaller the size of the generated particles, increasing lung deposition and reducing the nebulization time. High flows should be used to achieve particles of a size that is breathable (between 6 and 9 L/min). High-flow compressors (≥8 L/min) are recommended, however, to nebulize antibiotics, since they are more efficient and faster than conventional compressors, which generate a flow of 6–8 L/min.

**Figure 1:**
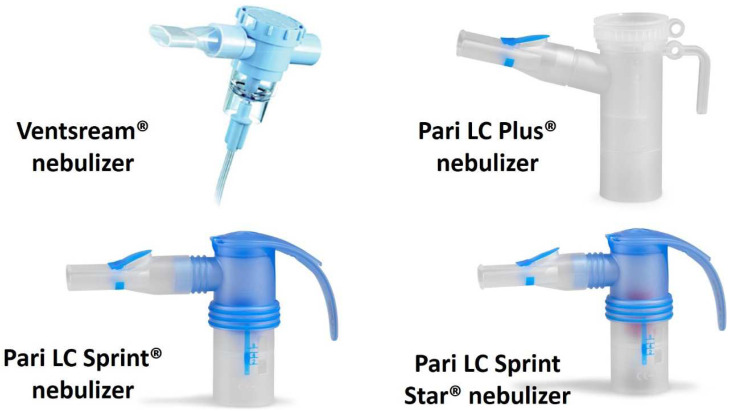
Jet nebulizers with an active Venturi effect during inspiration. Adapted with permission from: Máiz-Carro L, Martínez-García MA, de la Rosa-Carrillo D. Inhaled antibiotics; Neumología y Salud: Zaragoza, Spain, 2021, pp. 25–35 [19].

There are three types of jet nebulizers, classified according to their operation during inhalation:

1. Jet nebulizers with constant flow. These generate a continuous aerosol flow, but approximately 60–70% of the volume of the nebulized liquid is lost to the environment during the expiratory phase. This supposes both a loss of the nebulized drug and a risk of contaminating the environment, to the detriment of any people nearby.
2. Jet nebulizers with an active Venturi effect during inspiration. These nebulizers have a system that conducts the inspired air through the area that generates the aerosol, so that during the inspiratory phase the inspired flow is added to the flow generated by the compressor. Some jet nebulizers, such as the InnoSpire^®^, Pari LC Plus^®^, Pari LC Sprint^®^ and Pari LC Star^®^, also use a valve that closes the outlet during expiration, thereby reducing the loss of aerosol (Figure 1). These devices are more effective and faster than constant flow nebulizers [22].
3. Dosimetric jet or adapted aerosol release nebulizers. These systems release the aerosol according to the respiratory flow of each patient and deliver the aerosol only during inspiration. They are the most effective of the three, and they almost totally reduce the release of the nebulized drug into the environment [23].

To nebulize antibiotics with a jet nebulizer, a compressor with a high dynamic flow and a nebulizer that generates most of the respirable particles in the shortest possible time (10–15 min) should be used. The most recommended jet nebulizers are those with an active Venturi effect and a dosimetric capacity.

### 2.3. Mesh Nebulizers

**Figure 2:**
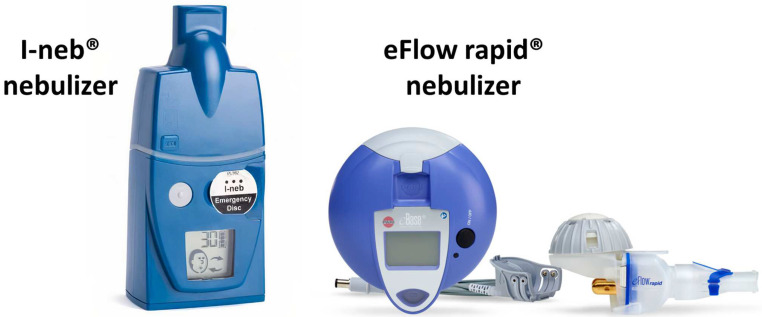
Mesh (electronic) nebulizers. Adapted with permission from: Máiz-Carro L, Martínez-García MA, de la Rosa-Carrillo D. Inhaled antibiotics; Neumología y Salud: Zaragoza, Spain, 2021, pp. 25–35 [19].

In this case, the aerosol is generated by passing the liquid to be nebulized through the holes in a mesh. Mesh nebulizers do not need a compressor and can be powered by electricity, batteries or a car battery. There are two main types: static and vibrating mesh nebulizers. In the former, the aerosol is generated by applying pressure to the liquid so that it passes through the holes in the mesh; in the latter, the liquid passes through the holes as a result of vibrations in the mesh. Mesh nebulizers are more effective than jet nebulizers, and they produce a greater lung deposition. Furthermore, they are smaller, lighter, quieter and faster [24] than jet nebulizers, resulting in better patient compliance. They also present a minimal residual dose and less waste of the drug [20,25]. The models most commonly used are those with vibrating mesh, such as the different eFlow^®^ models (Rapid^®^, Altera^®^, Tolero^®^, Zirela^®^) and the I-neb^®^ (Figure 2). The latter combines vibrating mesh technology with adapted aerosol release technology, only releasing the aerosol during the first phase of inspiration (50–80%) and thus avoiding aerosolization of the drug into the environment. Moreover, it continuously corrects the patient’s inhalation technique, warning and stopping if the nebulizer is not in the correct position, and it has the capacity to incorporate system recording compliance with treatment sessions.

Clinicians should be aware of the variability in the performance of different nebulizer systems. Technologies such as adaptive aerosol delivery and vibrating mesh technology have advantages over conventional systems in terms of treatment time, deposition as a percentage of priming dose, patient preference and adherence [26]. 

## 3. Dry Powder for Antibiotics

### 3.1. General/Technical Characteristics of the Devices

The earliest and still most widely used method of delivery of inhaled antibiotics is wet nebulization. In recent years, however, DPIs have been commercialized. These have several advantages over wet nebulizers: the administration time is much shorter, which can improve treatment adherence; they do not need time-consuming drug preparation or require maintenance, and they are small and portable. Furthermore, dry powder formulations are generally more stable than solutions, eliminating the need for cold chain storage and transportation. Since the inhalers are breath-activated, loss of the drug to the environment is minimal [27,28]. Although wet nebulizers can become contaminated if they are not cleaned according to instructions, DPIs do not require special cleaning or disinfection between uses (simply wiping the mouthpiece prior to administration with a dry cloth is sufficient) [29]. In contrast, DPIs have some drawbacks compared to nebulizers, such as some antibiotics are only available for nebulization, and DPIs generally require a proper inhalation technique and are therefore not suitable for all ages and patients. The forced inhalation required for DPI drug delivery is generally considered to be associated with a greater drug deposition in the oropharynx, compared to tidal breathing used for wet nebulization [30]. However, oropharyngeal deposition depends on many factors; it is not only related to the patient’s inspiratory flow, but also to upper airway anatomy, drug formulation and device resistance, among others. In some DPI antibiotics, the oropharyngeal impact is high and therefore its pulmonary deposit is highly variable. With others like TOBI Podhaler, it seems to be similar to what is produced with nebulizers, thanks to its formulation with PulmoSphere^®^ technology [31]. Moreover, microprocessor-controlled “smart” wet nebulizers, available for some antibiotics, use computer software that may be advantageous in some patients (i.e., for monitoring adherence to treatment) [32].

**Figure 3:**
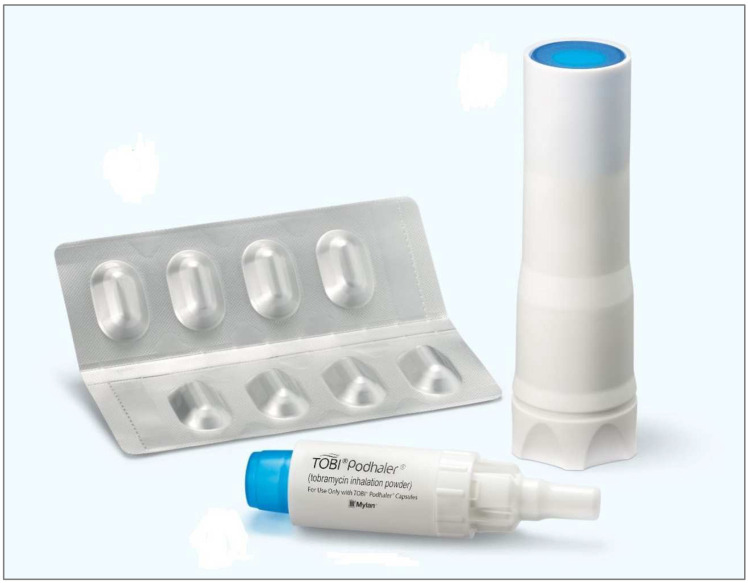
T-326 inhaler. Reprinted with permission from: E. Chiner Vives et al. Aerosolterapia. *Open Respir Arch.* **2020**, *2*, 89–99. 2021, Sociedad Española de Neumología y Cirugía Torácica (SEPAR) [35].

**Figure 4:**
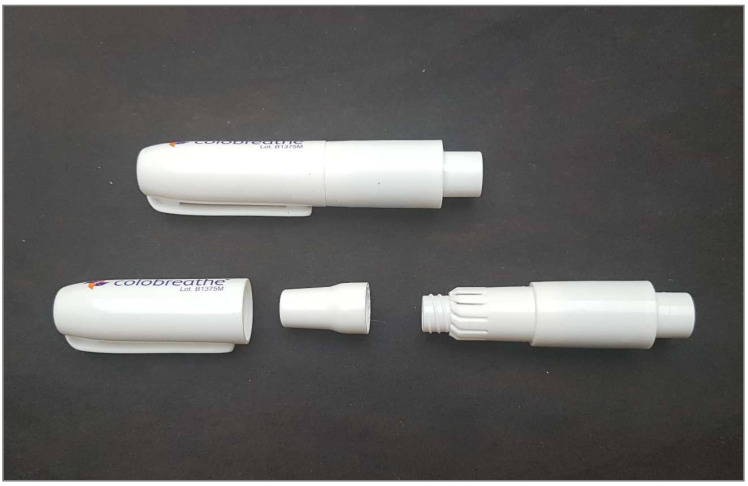
Turbospin inhaler. Photo taken by the authors.

There are currently two antibiotics available as DPIs: tobramycin (TOBI^®^ Podhaler; Novartis AG, Basel, Switzerland) and colistimethate sodium (Colobreathe^®^; Teva Pharmaceutical Industries, Tel Aviv, Israel). The development of ciprofloxacin (Ciprofloxacin Pulmosphere^®^; Bayer Pharma AG, Wuppertal, Germany), a drug with which phase 3 studies had already been carried out, has been discontinued. Commercially available tobramycin uses the T-326 inhaler (Novartis AG, Basel, Switzerland) (Figure 3), while colistimethate sodium uses the Turbospin™ device (PH&T, Milan, Italy) (Figure 4). Dry powder vancomycin was designed for use with the RS01 Model 7 device (Plastiape S.p.A, Lecco, Italy) but its development has been halted at the moment [33]. Other devices have been used with colistimethate sodium and tobramycin in some studies (Twincer™/Cyclops™, PureIMS, Roden, The Netherlands) [28,34]. The TOBI Podhaler uses the PulmoSphere™ (Novartis Pharmaceuticals, Basel, Switzerland) platform that creates spheroidal, highly porous particles. The TOBI^®^ Podhaler™ capsule-based drug and device combination is largely independent of flow rate, ranging from 48.9 to 88.4 L/min and inhaled volumes from 0.9 to 2.9 L, based on simulated patient breathing profiles, in a range of adult and paediatric anatomical throat models. The mean delivered dose ranged from 88.8% to 97.0% of declared capsule content, and the mean total lung dose across the range of flow profiles and anatomical throats tested was 63 ± 5% of the nominal dose [31].

The T-326 and Turbospin devices have a similar design and inhalation technique. The capsules containing the drug are stored in aluminium blisters that protect them from environmental humidity. The capsules are inserted into the device’s chamber, a spring-activated button is pressed to puncture the capsule and release the drug, and the patient inhales through the mouthpiece, causing the powder to be emptied into the chamber. The T-326 is a low-medium resistance device, and provided patients can generate an inhaled volume of greater than 1 L, 90% or more of the content of each capsule can be emptied with the first inhalation [36]. Studies with this device that included patients with forced expiratory volume in the first second (FEV_1_) as low as 25% of predicted value showed that all subjects achieved the required flow rates and that the majority reached an adequate inhalation volume [26], suggesting that the device is adequate even for patients with advanced lung disease, both in patients with CF and with bronchiectasis [37,38]. The aerosol performance of Colobreathe is poor compared to other DPIs, and is highly dependent on flow rate, with significant decreases in total lung dose with decreases in Q. This could lead to significant variability in lung dose due to differences in the anatomical features of the oropharynx among patients [39]. 

FEV_1_ is not a good predictor of the ability to reach an adequate inhalation volume, which is more related to the inspiratory capacity values [37]. Since it is impractical to measure inspiratory capacity in all cases, it is recommended that all patients perform two inhalation manoeuvres to ensure complete inhalation of the drug [37]. An additional inhalation manoeuvre should be performed if post-inhalation inspection of the capsule reveals that a significant amount of powder remains [30]. With Colobreathe^®^ 3–4 inhalations are needed by most patients to generate the 3.2 L of inhaled volume needed to empty the capsule [39]. Administration of the TOBI Podhaler requires inhalation of the contents of four capsules per dose. This requires less than 6 min, which is notably faster than nebulizing tobramycin solution [29].

Although safe and non-toxic [34], and designed to minimize the risk of fracture [27], the capsules containing the drugs may be shattered when pierced and this may be associated with throat irritation and coughing [40]. This is a more common problem with Colobreathe, as its hard gelatine capsules can fracture at the low humidity often required in packaging to maintain product stability. The TOBI Podhaler hydroxypropylmethylcellulose capsules retain their rheological properties at low humidity, making this effect less frequent. Patients and caregivers should be aware of this risk and should be instructed in the correct techniques to minimize it, which include piercing the capsule gradually and puncturing each capsule only once [40,41]. Despite these precautions, there is a feeling that there is a greater risk of a post-inhalation cough with dry powder inhalers than with nebulisation. However, this side effect appears to be more common with the Colistimethate Sodium DPI than with TIP [42,43]. In the case of TIP, the only study that has shown a higher frequency of cough with a DPI than with TOBI has been the EAGER study, since subsequent studies have not revealed a higher incidence of this adverse effect [44]. In any case, in those patients where a post-inhalation cough appears, it tends to disappear as treatment progresses. Some recommended measures to reduce coughing include drinking water between inhalations, taking shallower inhalations, improving inhalation technique and tilting the head back slightly during inhalation [45].

### 3.2. Deposition of Lung Particles

The deposition of lung particles using DPI antibiotics are crucial to any comparison of DPI antibiotics and nebulized antibiotics. If a treatment is to be efficient, it is important that the inhaled antibiotic can go through the upper airways and be deposited in the small airways. This important aspect is dependent on various particle-related factors and patient-related factors [46]. 

Particle-related factors include the shape, size, and density of the particles, which determine their aerodynamic behavior. The size distribution of an aerosol is described as the mass median aerodynamic diameter (MMAD), and most particles that make it into the small airways measure between 2 and 5 μm. Apart from size, particle density determines the velocity of transportation and the probability of deposition. Porous particles have a lower density than geometrically small non-porous particles, and this characteristic is exploited in the development of DPI drug formulations containing dry porous particles.

Patient-related factors with respect to lung particle deposition include the diameter of the airways, the presence of mucus in the airways, the quality of the inhalation manoeuvre and the lungs’ ability to expand. The presence of mucus, which is increased in bronchiectasis patients with chronic airway infection [47], can result in disturbance of the airflow pattern and thus increased deposition of antibiotic particles on obstructive sites. Another important point in bronchiectasis patients is the inhalation manoeuvre, which depends on the age, physical capability, disease severity and cognitive ability of the patient to perform a specific inhalation technique. In many DPIs, a rapid inspiratory flow will result in more turbulence in the central airways and more drug deposition in the upper airways [48]. However, as we have previously commented, rapid inspiratory flow is not the only factor that influences a greater or lesser lung and/or upper airway deposition, since there are more efficient inhalers. A slow inhalation manoeuvre, however, will result in less turbulence in the central airways and, therefore, a higher probability of aerosol lung particles bypassing the large central airways. Thus, the inhalation should, ideally, be slow and deep. 

Therefore, the selection of the most appropriate inhalation device to improve the deposition of lung particles should take into account not only the characteristics of the inhaled antibiotic but also the inhalation flow pattern, in relation to the device and the severity of disease. All these aspects must be considered whenever a DPI antibiotic is prescribed in patients with bronchiectasis and CBI.

### 3.3. Microbiological Issues

Airway bacterial load is a key component in the pathophysiology of CBI in patients with bronchiectasis [49] and other chronic inflammatory diseases [5,50]. It has been demonstrated that a high bacterial load is associated with increased local and systemic inflammation, more severe disease and poorer clinical outcomes. Moreover, post-hoc analyses have demonstrated that a high bacterial load is a consistent predictor of the inhaled antibiotic response, as patients with an elevated baseline bacterial load had a clear improvement in respiratory symptoms [51]. One of the targets of inhaled antibiotic treatment is, therefore, to decrease the airway bacterial load. This is despite no study having shown that there is a direct relationship between the reduction of bacterial load or microbiological eradication with the use of DPI antibiotic therapy, and an improvement in exacerbations.

A recent real-life study showed that treatment with DPI antibiotics reduced the purulence and quantity of sputum and the microbiological load [43]. In one multi-centre Spanish study in which 164 bronchiectasis patients on DPIs were included, a significant reduction was observed in the presence of CBI by both *P. aeruginosa* and other PPM, as well as in sputum production and the purulent or mucopurulent sputum aspect, in a comparison between data one year prior to prescription and those one year after prescription. Furthermore, a reduction was observed in exacerbations (non-severe and severe) and hospitalizations. Most of the patients had received the colistimethate dry powder compared to the tobramycin dry powder, although no clinical and microbiological differences between the two treatments were found.

The emergence of resistant bacterial strains is another important microbiological issue in patients receiving inhaled antibiotics [52]. It has traditionally been assumed that the chronic exposure of a pathogen to a level of a specific antibiotic insufficient to eradicate that microorganism will lead to the development of resistant strains. However, most of the studies performed in patients with bronchiectasis (both nebulized and DPIs) have not shown any increase in the frequency of antimicrobial-resistant isolates [53]. The reasons for this lack of resistance have not been fully studied but they are likely to be multifactorial. A minimum inhibitory concentration (MIC) for each antibiotic is usually determined via parenteral breakpoints, but it is not clear whether these would be applicable in the case of inhaled antibiotics, which lead to higher sputum concentrations than those achieved by antibiotics administered parenterally with no toxic effects. As the use of inhaled antibiotics will be further explored in the near future, it would be extremely interesting to determine an airway-adjusted MIC that corresponds more directly with the concentrations achieved using the inhaled route.

## 4. Studies with Inhaled Dry Powder Antibiotics in Cystic Fibrosis Patients

### 4.1. Tobramycin Studies

Three studies have evaluated the efficacy and safety of tobramycin-inhaled dry powder (TIP) in stable CF patients chronically infected by *P. aeruginosa*: EVOLVE, EDIT and EAGER (Table 2 [54]). The EVOLVE [16] and EDIT [55] trials evaluated the efficacy and safety of TIP compared to placebo, and EAGER [56], which included the largest number of patients, determined non-inferiority against tobramycin inhalation solution (TIS) after 3 treatment cycles. The dose of TIP used in the three studies was 112 mg twice daily (4 × 28 mg capsules) in 28-day “on” cycles followed by 28 days of “off” cycles. The mean TIP administration time was 4 to 6 min. In all three trials, efficacy was assessed primarily on the basis of relative changes in FEV_1_ from baseline in each group and reduction in *P. aeruginosa* density, as well as the need for antipseudomonal antibiotics. 

The EVOLVE study reported that the *P. aeruginosa* sputum density was more commonly reduced with TIP than placebo, but an increasing trend of resistance to tobramycin compared to baseline was observed in the group of patients treated with TIP (although this trend was also found in the placebo group). Similarly, a significant improvement in predicted FEV_1_% was also observed. The proportion of patients requiring any additional antipseudomonal antibiotic in cycle 1 was lower with TIP than with placebo (13.0% vs. 18.4%), and the duration of these treatments was shorter in the TIP group.

In the EAGER study, there were no significant differences between TIP and TIS in the mean reduction in sputum *P. aeruginosa* density and in increases in FEV_1_%. Administration time was significantly shorter for TIP. Treatment satisfaction was estimated using a version of the Treatment Satisfaction Questionnaire for Medication; patients treated with TIP were significantly more satisfied. However, the proportion of patients requiring any new antipseudomonal antibiotic was significantly higher with TIP than with TIS (64.9% versus 54.5%), although the average number of antibiotic days tended to be less in the TIP group. The efficacy of TIP was sustained for up to seven cycles, and long-term treatment with TIP was generally safe and well tolerated, with no increase in adverse events [57]. 

A post-hoc analysis of the EAGER trial evaluated the safety and efficacy of TIP vs. TIS in different age groups [58]. The mean difference in FEV_1_% between TIP vs. TIS decreased with age, and no differences were observed with respect to *P. aeruginosa* density in the different age groups. People of all ages reported greater convenience with TIP compared to TIS, and, furthermore, adolescents and adults treated with TIP were more satisfied with the effectiveness of the treatment. More individuals treated with TIP reported cough and dysphonia, while more children and adolescents treated with TIS reported upper respiratory tract infection. Another secondary analysis of the EAGER study explored the economic value of DPIs over the aerosolized formulation. TIP was associated with lower costs and greater quality-adjusted-life-years than TIS [59].

Most of the adverse events observed with TIP were transient and mild to moderate, cough being the most frequent, although it seemed to disappear with successive cycles. Dysphonia and dysgeusia were also more common and, among the most serious adverse effects, it is worth mentioning pulmonary exacerbations and bronchospasm, although they were not more frequent than they were with placebos or with TIS [16,55,56]. 

Several studies such as ETOILES [60], Greenwood et al. [61] and FREE [62] have been conducted to collect real-life data to investigate whether the features of TIP explain the actual benefits found in clinical practice in previous research. Results from these studies showed that TIP treatment was associated with benefits to lung function and reductions in the sputum density of *P. aeruginosa*, without any further increase in the MIC (minimum inhibitory concentration). Greenwood et al. showed that the T-326 inhaler used to administer TIP was easy to use and required a shorter total administration time, compared to the nebulizers used to administer TIS and colistin. In another real-life study, Greenberg et al. assessed patients’ treatment satisfaction with TIP, in a routine clinical setting, using a web survey, including the Treatment Satisfaction Questionnaire for Medication [63]. The majority expressed satisfaction with TIP’s administration time, cleaning time, portability and ease of use.

### 4.2. Colistin Studies

The Freedom Study was an active, randomized, open-label study that evaluated whether the colistimethate DPI over a 24-week period was not inferior to TIS in 380 CF patients with CBI by *P. aeruginosa* in terms of loss of respiratory function, antibiotic sensitivity or side effects. The exacerbation rate was not taken into account to determine the effectiveness of either drug [17]. After the completion of a minimum of two cycles of TIS, patients were then randomized for continuous treatment over a 24-week period with Colobreathe^®^ (one capsule of 1,662,500 IU twice daily) or for three 28-day courses of TIS (300 mg/5 mL tobramycin, twice daily) using a PARI LC Plus nebulizer with a specific compressor. Each TIS course was followed by a 28-day “off” period. The study’s results showed that the colistimethate DPI was not inferior to TIS for the treatment of CBI by *P. aeruginosa* in CF patients in terms of changes in FEV_1_%, adverse events and the susceptibility of *P. aeruginosa* isolates to colistin. The proportion of colistin-resistant isolates in both groups was low (≤1.1%) and did not increase during the study. A significantly higher proportion of patients rated the Turbospin device as ‘easy or very easy to use’ (90.7%), when compared with patients in the TIS group (53.9%). There was a higher occurrence of cough (75.4% vs. 43.5%), throat irritation (45.5% vs. 28.0%) and abnormal taste (62.6% vs. 27.5%) in the colistimethate DPI group than in the TIS group. Most side effects were mild to moderate and were resolved without any sequelae. Discontinuations due to an adverse event were also higher in the DPI group (9.7% vs. 1.6%). 

As regards side effects, a more recent cross-sectional study from Kaplan et al. evaluated information about the correct use of Turbospin and the adverse effects derived from the colistimethate DPI. Separate survey questionnaires were developed for healthcare professionals (HCPs), patients and patients’ caregivers from Austria, Denmark, France, Germany, the Netherlands and the United Kingdom (UK). A total of 124 subjects participated between September 2016 and March 2018. Most of the HCPs and patients/caregivers who participated in this study had a good knowledge of common side effects associated with the colistimethate DPI and of its correct use, but their knowledge of the correct use of the Turbospin inhaler and possible capsule breakage was moderate to low. These results should be interpreted with caution, however, due to the small number of patients/caregivers who participated in this study [41].

Another study from the same authors, carried out with data obtained from the United Kingdom CF patient registry from 2014 to 2018, analyzed adverse effects in 1466 patients who had received the colistimethate DPI, and compared them with a cohort of 3503 patients who had received other inhaled antibiotics. The primary outcome was a composite endpoint, defined as adverse events or new CF complications. Other outcomes included pulmonary exacerbations and discontinuations of treatment. There was no difference in the rate of adverse events between the colistimethate DPI cohort and the other one. The rates of discontinuation were similar in the colistimethate DPI and in the TIP cohorts (37.8% and 39.8% of patients, respectively). The authors concluded that the safety profile of the colistimethate DPI is similar to that of other inhaled antibiotics, thereby endorsing its long-term safety in patients with CF [42].

Finally, we also highlight two studies whose results have not been published, although the manufacturer sent to them to NICE. The COLO/DPI/02/06 trial was an open-label, non-inferiority trial that was carried out in 66 centres in European Union countries, Russia and Ukraine. It compared the efficacy and safety of continuous treatment with the colistimethate sodium DPI and intermittent (28 days on/off) nebulized TIS over 24 weeks. It concluded that the colistimethate sodium DPI was not inferior to nebulized tobramycin as it met the predefined non-inferiority criteria. Another much smaller (*n* = 16) trial, COLO/DPI/02/05, was conducted in three centres in the UK to compare colistimethate sodium DPI with nebulized colistimethate. It was a crossover trial reporting outcome data at 4 weeks (before crossover) and 8 weeks only (after crossover) and study data were not published [64].

In summary, the effectiveness of the colistimethate DPI administered by the Turbospin^®^ inhaler was not inferior to TIS for the treatment of CBI in CF patients, and it was well tolerated, with a similar profile to that of TIS, although coughing and bad taste were very common adverse effects. The ease and speed of use of the colistimethate DPI could have a positive impact on compliance.

## 5. Studies with Antibiotic Dry Powder Inhalers in Bronchiectasis

Colistin and tobramycin are the most widely used inhaled antibiotics in bronchiectasis [65]. As mentioned above, both dry powder tobramycin and colistimethate have demonstrated good efficacy and safety in some clinical trials conducted in patients with CF and CBI by *P. aeruginosa* [30,64,66,67]. This efficacy, together with the advantages of DPIs over nebulizers, have led to their increasing use in other airway diseases that cause CBI, especially in patients with bronchiectasis of other aetiologies. Although there have been some prospective observational and RCT studies on the efficacy and safety of colistin or tobramycin in bronchiectasis [68,69,70], to date there has been only one randomized clinical trial with the DPI formulation of these drugs: the iBest Study. This phase II, double-blind study aimed to determine the efficacy and safety of different dosages of TIP in patients with non-CF bronchiectasis. One hundred and seven patients with bronchiectasis and chronic *P. aeruginosa* infection were included and randomized 1:1:1 to three different doses of TIP for 16 weeks and followed for 8 weeks. All three doses of TIP significantly reduced sputum density of *P. aeruginosa* compared with a placebo in a dose-dependent manner, and a smaller proportion of patients in any of the TIP groups experienced pulmonary exacerbations compared with the placebo, although not significantly. Some patients (23.4%) discontinued the study due to adverse events. Outside of this trial, the only other reported clinical trials with dry powder drugs (beyond bronchodilators and/or inhaled corticosteroids) in bronchiectasis patients were conducted with ciprofloxacin (antibiotic) [71,72,73,74,75] and mannitol (non-antibiotic) [67]. There have also been two observational studies on the efficacy and safety of dry powder tobramycin and colistimethate in bronchiectasis [31,46].

In a small observational study, eight patients with bronchiectasis were included and treated with a formulation of dry powder tobramycin free base (which is different from the tobramycin sulphate inhalation powder administered with the Podhaler) administered using the Cyclops DPI without excipients, in order to assess the local tolerability and pharmacokinetics at doses of 30, 60, 120 and 240 mg per day. The study demonstrated that this tobramycin DPI formulation was well tolerated at these doses. Only two patients reported coughing, which disappeared during follow-up, and in four patients the FEV_1_ decreased by more than 10% (which could be reversed by using a bronchodilator) after inhaling tobramycin, although these patients were finally diagnosed with asthma and did not present any additional increase in dyspnoea [34]. However, in this case, questions would arise regarding the long-term safety of the use of tobramycin free base, which has a pH of approximately 10 in water with significant buffering capacity due to its five primary amines.

Furthermore, a real-life, observational, multi-centre and retrospective study (historical cohorts) evaluated the efficacy and safety of dry powder tobramycin and colistimethate by inhalation in Spain in 161 patients with bronchiectasis recruited from 33 centres [43]. The patients had a mean age of 65.7 years, and 37% had concomitant COPD and 20.6% had asthma. The indication for the dry powder antibiotic was CBI due to *P. aeruginosa* in 86% of the cases, and in most cases (65.1%) these patients had taken a previous inhaled antibiotic that was poorly tolerated or ineffective. Comparing the year before the start of treatment with the year after that, the efficacy of the DPI antibiotic was generally excellent, with a significant reduction in the number of exacerbations, both non-severe (1.9 vs. 1.77; *p* = 0.023) and especially severe (0.73. vs. 0.33; *p* < 0.0001), with a reduction of more than 50% of patients defined as exacerbators. From a microbiological point of view, the finding of CBI by *P. aeruginosa* decreased from 81% to 52% of patients, while the CBI by other PPMs decreased from 29.4% to 10%, without any changes in the fungal or atypical mycobacteria isolations. Similarly, there was a decrease in both daily sputum production and its purulence, although no significant changes were observed in dyspnoea or lung function. Finally, although the number of patients treated with TIP was low (only 14%), no significant differences were observed in terms of efficacy, compared with dry powder colistimethate.

As regards safety concerns, a high percentage of patients (40.8%) presented with a persistent cough. The profile of these patients with a cough was older, with a longer time since the diagnosis of bronchiectasis, a presence of associated COPD, previous cough, previous exacerbations and greater difficulty in handling the inhalation device. As a result, 24.4% had to withdraw treatment, especially because of persistent cough. The patients who had to abandon treatment were those who received less information from the physician or nurse on how to use the device, less training in its use and no administration of the first dose in a hospital. With respect to the comparison between the different antibiotics, those patients who took colistimethate had fewer withdrawals from treatment (20% vs. 27%; *p* = 0.042), less difficulty in using the device and a lower proportion of resistance to *P. aeruginosa* (3% vs. 22%; *p* = 0.001). Therefore, this study showed that DPI antibiotics are effective in patients with bronchiectasis, although they present a significant number of adverse effects, mainly a persistent cough, especially in patients with a more severe disease, of an older age or associated respiratory comorbidities. Moreover, the study also revealed that information and instructions from health personnel to the patient on how to use the device are crucial to achieving a reduction in adverse effects. Given the results of these studies, it is necessary to carry out clinical trials on the efficacy and safety of colistin and tobramycin in dry powder in patients with bronchiectasis. One of the issues that clinicians have been most concerned about with the use of DPI antibiotics in CF is a possible increased risk of local adverse effects, especially persistent cough, compared with the administration of antibiotics by nebulizers. These adverse effects could be more substantial in patients with bronchiectasis, given their different profile compared to those with CF (older age, a greater number and severity of respiratory comorbidities such as COPD and asthma and poorer baseline lung function) [76,77]. However, as we have commented, even in these patients the side effects of DPI antibiotics are less frequent than feared, and usually mild or moderate. Furthermore, in many cases they depend on factors related to the galenic of the pharmacological presentation, such as the drug dose, the amount of powder in each capsule, the pH of the preparation or the osmolality of the formulation, among others. These conditioning factors are more closely related to certain drugs, although it is to be expected that in the future these tolerance problems can be corrected with new formulations.

## 6. Other Dry Powder Antibiotics

### 6.1. Anti-Tuberculous Agents

The treatment of tuberculosis is complex. It requires multiple drugs for a prolonged period of time and a considerable rate of antibiotic resistance has been identified. Several efforts have been made to evaluate inhaled anti-tuberculous drug powders in order to improve current treatment options. So far, however, primary alveolar macrophages, the most important cells for *Mycobacterium tuberculosis* infection, have not proved easily accessible [78]. Most of this research has been conducted in a pre-clinical setting and assessed the pharmacokinetics, safety and efficacy of inhaled formulations in animal models. In this regard, Verma et al. showed that clofazimine as a dry powder microparticle formulation for inhalation not only had a similar fatal capacity against *M. tuberculosis* as the native compound in vitro, but also reduced colony forming units (CFU) in the lungs of mice, compared to the oral formulation in vivo [79]. 

Furthermore, Parikh et al. compared the uptake of rifampicin microparticles by alveolar macrophages in mice, whether administered orally or by the intratracheal route. They observed a significantly higher concentration of the drug in alveolar macrophages, as well as a higher production of mycobactericidal nitric oxide after intratracheal administration. These findings led the authors to conclude that dry powder inhalation of rifampicin may reduce the treatment time of tuberculosis and lower the chances of developing drug resistance [80]. 

Another study compared the biodistribution and pharmacokinetics of isoniazid and rifabutin administered as a DPI to rhesus macaques. The authors showed that concentrations of isoniazid and rifabutin in the lungs were twice that of the liver and four times that of the kidneys. Moreover, the elimination and half-life of both drugs were higher when they were inhaled, compared to intravenous administration, suggesting that DPIs would improve the biodistribution of these agents in human patients [81]. More recently, Chogale et al. investigated nanoformulations of isoniazid, pyrazinamide and rifampicin combined as a DPI, showing a prolonged lung deposition compared to oral therapy. These could complement existing anti-tuberculous therapy and enhance the efficacy of treatment [82]. 

Only limited data on clinical trials of inhaled anti-tuberculous treatments are currently available. A phase I study by Dharmadhikari et al. examined the safety and tolerability of a single-dose DPI of capreomycin in 20 healthy subjects. Inhaled capreomycin was well-tolerated and rapidly absorbed, achieving serum drug concentrations above the minimum inhibitory concentrations (MIC) for *M. tuberculosis* [83]. A study by Srichana et al. evaluated the sequential challenge of dry powder formulations of isoniazid, rifampicin, pyrazinamide and levofloxacin in different orders, in 40 healthy volunteers. No significant increases in pro-inflammatory cytokines in sputum or deterioration of lung function parameters, or any other adverse events, were observed, leading the authors to propose that the inhaled administration of these drugs is safe [84]. In this respect, one randomized, placebo-controlled trial compared the efficacy and safety of a DPI combination of isoniazid, rifampicin, pyrazinamide and levofloxacin to a placebo in 91 patients diagnosed with pulmonary tuberculosis. The authors showed a trend towards an earlier sputum culture conversion in the study group, although this was not statistically significant. Additionally, they observed a significantly lower proportion of cough and a lower incidence of nausea and vomiting in the study group, with no relevant adverse events [85]. Overall, these findings indicate a great potential for inhaled therapy as a part of anti-tuberculous treatments.

### 6.2. Ciprofloxacin

A phase I, randomized, dose-escalation study including 25 CF patients compared the ciprofloxacin DPI to a placebo. The authors showed a rapid absorption of the drug and no serious treatment-emergent adverse events. Furthermore, minimal systemic exposure was observed, while the sputum concentrations of ciprofloxacin were over 100-times the MIC for *P. aeruginosa*, much higher the usual measurements after oral administrations [71]. In a phase II study, 124 patients with culture-positive bronchiectasis were randomly assigned to receive either DPI ciprofloxacin or placebo for 28 days. Subjects in the treatment group showed a significant reduction in sputum bacterial load at the end of the study, but this went back up again in the follow-up period. Moreover, 35% of treated patients achieved pathogen eradication, versus only 8% in the placebo group [64]. The most frequently described treatment-emergent adverse event was dysgeusia, and the rates of bronchospasm and cough were low [71,72].

Recently, two large phase III trials in bronchiectasis patients have been completed, the RESPIRE 1 and RESPIRE 2 trials. Participants were required to have a history of two or more exacerbations in the previous year and predefined bacteria in sputum. In both studies, patients were randomized to 14- and 28-day on/off regimens of the ciprofloxacin DPI or a placebo. Although a statistically significant prolongation of the time to the first exacerbation and a reduction in the frequency of exacerbations were observed in the ciprofloxacin DPI 14-day arm of the RESPIRE 1 study, these findings were not replicated in either the 14- or 28-day treatment arms of the RESPIRE 2 trial [74,77]. These clinical trials were designed to include patients with a minimum of two respiratory exacerbations in the previous year, but the number of exacerbations in both placebo groups was much lower. In the RESPIRE –2 study, the mean number of exacerbations in the overall number of included patients was 0.6 during the 48-month follow-up. This notably affected the results, causing the primary objective, which was the reduction of exacerbations, not to be achieved. It is also worth noting that the rate of respiratory side effects (cough, breathlessness, bronchospasm) was very low in both clinical trials. It is very striking that they were also significantly less frequent than the adverse effects in the ORBIT 3 and ORBIT 4 clinical trials [86], carried out with a nebulized liposomal formulation of ciprofloxacin. This indicates that the presentation of a cough and other adverse effects observed with some presentations of DPI antibiotics is more related to the formulation of the drug (pH, excipients, etc.), than to the fact that it is a powdered product [87]. Overall, the ciprofloxacin DPI is safe and well tolerated, and could potentially be a good treatment option for both CF and selected bronchiectasis patients.

### 6.3. Meropenem

Muneer et al. developed a DPI formulation of meropenem using lactose as a carrier alone or in combination with L-leucine and magnesium stearate [88]. The authors reported that the fine particle fraction (FPF) of this formulation was similar to that of other DPI presentations of antibiotics on the market and that it showed a great potential for delivery to the lungs.

### 6.4. Doxycycline

Experimental research is underway to develop new treatments in DPI formulations. In this regard, Douafer et al. have studied the combination of doxycycline with an adjuvant (polyaminoisoprenyl derivative NV716) for the treatment of *P. aeruginosa* infections. This adjuvant enables the restoration of antibacterial activity against naturally resistant strains due to outer-membrane impermeability. The authors demonstrated that the inhaled antibiotic/adjuvant dry powder combination is viable and could help to improve the efficacy of treatment [89].

### 6.5. Vancomycin

Methicillin-resistant *Staphylococcus aureus* (MRSA) is a significant pathogen in patients with chronic airway disease. Classic treatment with vancomycin administered intravenously is effective, but its penetration into pulmonary secretions is poor, and it also causes notable renal toxicity that limits the dose and makes it less effective in the respiratory environment. Under the premise that inhaled administration of vancomycin would overcome these limitations, a DPI formulation was developed (AeroVanc, Savara Inc., Austin, TX, USA). The phase 1 study aimed at demonstrating safety and tolerability was carried out in healthy subjects and patients with CF, who received different doses of the drug in a single administration [90]. There were no serious side effects: vancomycin plasma concentrations were proportional to the administered dose and the mean sputum vancomycin trough concentration remained above the usual MRSA MIC values for up to 24 h. With these promising results, the phase 2 study was launched in 87 patients with CF and CBI by MRSA [91]. They received AeroVanc at two different doses or placebo, twice daily, for a period of 28 days. No differences were observed in the frequency of adverse reactions, which were mild, and AeroVanc-treated patients had a statistically significant reduction in sputum MRSA density compared with placebo-treated subjects. According to the company, secondary endpoints, including improvement in lung function, time to exacerbation and reduction in respiratory symptoms, “showed encouraging trends.” These results prompted the launch in 2017 of the phase 3 trial (AVAIL trial), which included 188 CF patients [92]. They received AeroVanc 30 mg twice daily during the 24-week period vs. placebo, over three dosing cycles, each cycle was 28 days of treatment followed by 28 days of observation. Subsequently, all the patients passed to the open-label period 2, at the same doses and with the same frequency of administration. However, the trial failed to show any statistically significant improvement in lung function or any reduction in exacerbations, so the company decided to discontinue development of the drug in December 2020.

## 7. Conclusions

The use of inhaled antibiotics offers the possibility of directly treating patients with chronic airway diseases and recurrent infections, minimizing the adverse effects of systemic antibiotic therapy. Until recently, the only option for this was nebulization using jet devices, which required a lot of time to prepare and administer each dose. The advent of more modern nebulizers, such as mesh nebulizers, reduced the nebulization time, but it was still necessary to prepare the doses and, above all, to clean and sterilize the equipment after each use. All of this greatly affects the quality of life of patients who suffer from chronic bronchial infection and require this type of treatment.

The development of dry powder inhalers has meant a great advance in the treatment of the most prevalent respiratory diseases, such as asthma and COPD, by facilitating the administration of drugs and reducing the usual errors with pMDIs. In the specific case of inhaled antibiotics, they represent a notable advance, by reducing the administration, preparation and cleaning time associated with the use of nebulizers although a good previous educational and physiotherapy programme would be very important to maximize the tolerability of dry powder inhaled antibiotics [93]. Furthermore, published studies, despite their limitations, show that their effectiveness and safety are comparable to those of nebulized administration. All of these findings entail notable benefits for patients, especially those who are socially and occupationally active, allowing them to lead a more normal life. 

## Figures and Tables

**Table 1:** Main characteristics of the different types of drug nebulization systems.

	Advantages	Drawbacks
Ultrasonic nebulizers	They allow large volumes of liquid to be nebulized. They are silent.	They produce highly heterogeneous and dispersed aerosols. Some drugs are denatured by heat (for example, antibiotics). They do not nebulize suspensions.
Jet nebulizers	They provide high flows. They are faster than ultrasonic nebulizers. They can nebulize both solutions and suspensions.	Their compressors are usually loud and heavy.
Mesh nebulizers	Some can run on batteries (as well as electricity). They are small and silent. They can nebulize both solutions and suspensions. They are faster than jet nebulizers.	They are less resistant than jet nebulizers. There is a lack of bioequivalence studies with jet nebulizers for some drugs.

Adapted with permission from: Máiz-Carro L, Martínez-García MA, de la Rosa-Carrillo D. Inhaled antibiotics; Neumología y Salud: Zaragoza, Spain, 2021, pp. 25–35 [19].

**Table 2:** Main studies with tobramycin dry powder inhaler.

	EVOLVE Study	EDIT Study	EAGER Study
Study design	Randomized, double blind, compared with placebo. TIP: 112 mg bid (*n* = 46); placebo (*n* = 49) 24 weeks (1 cycle TIP or placebo followed by 2 cycles open-label TIP). To evaluate the efficacy of a 28-day bid dosing regimen of TIP vs. placebo, as measured by the relative change in FEV1% predicted from baseline to the end of the dosing in cycle 1.	Randomized, double blind, compared with placebo. TIP: 112 mg bid (*n* = 30); placebo (*n* = 32) 8 weeks (1 cycle TIP or placebo). Extensions: Each extension consisted of 3 additional cycles of TIP. To evaluate the efficacy of TIP manufactured by an improved process vs. placebo, assessed by relative change in FEV_1_% predicted from baseline to day 29.	Randomized, open label, non-inferiority. TIP: 112 mg bid (*n* = 308); TIS: 300 mg/5 mL bid (*n* = 209) 24 weeks (3 cycles TIP or TIS. To evaluate the safety of bid dosing of TIP delivered with the T-326 inhaler vs. TIS (5 mL) delivered with the PARI-LC^®^ PLUS Jet nebulizer.
FEV_1_	Baseline to day 28-TIP vs. placebo: 13.3% (95% CI: 5.3 to 21.3; *p* = 0.0016).	Baseline to day 29-TIP vs. placebo: 5.9% (95% CI: −2.2 to 14.0; *p* = 0.148).	Baseline to day 28 of cycle 3-TIP vs. TIS: 1.1% relative change (least squares mean difference).
Antibiotic use	TIP vs. placebo: 13.0% vs. 18.4%.	TIP vs. placebo: 6.7% versus 12.5%.	TIP vs. TIS: 64.9% vs. 54.5%.
*P. aeruginosa*	Non-mucoid PA-TIP vs. placebo: −1.91 (SD: 2.54) vs. −0.15 (0.68). Mucoid-TIP vs. placebo: −2.61 (2.53) vs. −0.43 (1.05).	Sum of all biotypes PA-TIP vs. placebo): −1.2 vs. 0 (*p* = 0.002).	Nonmucoid PA-TIP vs. TIS: −1.77 vs. −0.73. Mucoid-TIP vs. TIS: −1.6 vs. −0.92.
Overall safety	TIP vs. placebo: 23 (50.0%) vs. 37 (75.5%).	TIP vs. placebo: 8 (26.7%) vs. 11 (34.4%).	TIP vs. TIS: 278 (90.3%) vs. 176 (84.2%).
Cough	TIP vs. placebo: 6 (13.0%) vs. 13 (26.5%)	TIP vs. placebo: 3 (10.0%) vs. 0	TIP vs. TIS: 149 (48.4%) vs. 65 (31.1%)
Suspension	TIP vs. placebo: 7 (15.2%) versus 9 (18.4%).	TIP vs. placebo: 1 (3.3%) vs. 1 (3.1%).	TIP vs. TIS: 83 (26.9%) vs. 38 (18.2).

TIP: Tobramycin inhalation powder. TIS: Tobramycin Inhalation Solution. FEV_1_: Forced expiratory volume in the first second. PA: *Pseudomonas aeruginosa*. Modified from: Hamed K, et al. *Ther. Adv. Respir. Dis.* **2017**, *11*, 193–220. Reprinted by Permission of SAGE Publications [54].

## Data Availability

Data sharing not applicable. No new data were created or analyzed in this study. Data sharing is not applicable to this article.
